# Biodiversity of Philippine marine fishes: A DNA barcode reference library based on voucher specimens

**DOI:** 10.1038/s41597-023-02306-9

**Published:** 2023-06-24

**Authors:** Katherine E. Bemis, Matthew G. Girard, Mudjekeewis D. Santos, Kent E. Carpenter, Jonathan R. Deeds, Diane E. Pitassy, Nicko Amor L. Flores, Elizabeth S. Hunter, Amy C. Driskell, Kenneth S. Macdonald, Lee A. Weigt, Jeffrey T. Williams

**Affiliations:** 1 National Systematics Laboratory, Office of Science and Technology, NOAA Fisheries, Washington, D.C. 20560 USA; 2 Department of Vertebrate Zoology, National Museum of Natural History, Smithsonian Institution, Washington, D.C. 20560 USA; 3 Biodiversity Institute, University of Kansas, Lawrence, Kansas 66045 USA; 4 Genetic Fingerprinting Laboratory, National Fisheries Research and Development Institute, Quezon City, 1103 Philippines; 5 Department of Biological Sciences, Old Dominion University, Norfolk, Virginia 23529 USA; 6 Center for Food Safety and Applied Nutrition, United States Food and Drug Administration, College Park, Maryland 20740 USA; 7 Laboratories of Analytical Biology, National Museum of Natural History, Smithsonian Institution, Washington, D.C. 20560 USA

**Keywords:** Ichthyology, DNA sequencing

## Abstract

Accurate identification of fishes is essential for understanding their biology and to ensure food safety for consumers. DNA barcoding is an important tool because it can verify identifications of both whole and processed fishes that have had key morphological characters removed (e.g., filets, fish meal); however, DNA reference libraries are incomplete, and public repositories for sequence data contain incorrectly identified sequences. During a nine-year sampling program in the Philippines, a global biodiversity hotspot for marine fishes, we developed a verified reference library of cytochrome c oxidase subunit I (COI) sequences for 2,525 specimens representing 984 species. Specimens were primarily purchased from markets, with additional diversity collected using rotenone or fishing gear. Species identifications were verified based on taxonomic, phenotypic, and genotypic data, and sequences are associated with voucher specimens, live-color photographs, and genetic samples catalogued at Smithsonian Institution, National Museum of Natural History. The Biodiversity of Philippine Marine Fishes dataset is released herein to increase knowledge of species diversity and distributions and to facilitate accurate identification of market fishes.

## Background & Summary

In 2007, the United States Senate requested the Government Accountability Office (GAO^1^) investigate several federal agencies responsible for seafood and determine actions necessary to detect and prevent seafood fraud. GAO (2009) found that Americans consumed almost 5 billion pounds of seafood annually, more than 80% of which was imported, and determined that current practices were not sufficient to assure consumers that the seafood they purchased was correctly labeled. The Presidential Task Force on Combating Illegal, Unreported, and Unregulated (IUU) Fishing and Seafood Fraud identified products like grouper and snapper as particularly at risk because the same species are marketed under different names in different countries, and extensive processing, common for imported products, removes diagnostic characters required for morphological species identification^2^. As part of its summary report, GAO^1^ recommended “a federal agency wide library of seafood species standards” be developed. In response, the United States Food and Drug Administration (FDA), which is responsible for the identification of seafood distributed through interstate commerce, established a collaboration with the Smithsonian Institution’s National Museum of Natural History (NMNH), Division of Fishes and Laboratories of Analytical Biology (LAB) to develop a DNA-barcode reference library for commercial seafood as a regulatory tool for species identification^3–5^.

During the past 30 years, genetic sequencing has improved the identification of global biodiversity. Commonly known as DNA barcoding, sequencing one or more loci and comparing these data to those in reference libraries is an efficient way to identify or verify species. DNA barcoding has been used to identify species diversity of a region (e.g.^6–9^), describe new species (e.g.^10^), link different ontogenetic stages (e.g.^11,12^), detect illegally traded wildlife (e.g.^13^), and confirm the identity of animals sold in markets (e.g.^14^). The FDA now uses DNA barcoding as its primary tool for species identification of seafood products and has used this technique successfully both in seafood-related-illness investigations^15,16^ and in cases of species substitution for economic gain^17^.

Success of species-level identification using barcodes depends on completeness and accuracy of DNA reference libraries^8,18,19^. Public sequence repositories such as GenBank (https://www.ncbi.nlm.nih.gov) and the Barcode of Life Database (BOLD; https://www.boldsystems.org/) are essential resources for DNA barcoding, yet they contain misidentified sequences (e.g.^20–23^). Misidentifications can happen because some species are difficult to identify morphologically or because of errors in data management. In other cases, sequence data does not align with currently accepted morphological identifications for legitimate reasons including cryptic diversity, mixed genealogies between sister species, incomplete lineage sorting, and introgressive hybridization^8^. Incongruent sequences that lack associated voucher specimens, have incomplete collection data, or lack protocols for revising identifications can make problematic sequences in public repositories difficult to resolve. Thus, reference libraries greatly increase in scientific and practical value when sequences are linked to cataloged voucher specimens in natural history museums that can be reexamined to verify or revise identifications.

With more than 7,600 islands and 36,000 kilometers of coastline in the Coral Triangle, the Philippines is the epicenter of marine shorefish biodiversity and home to more than 2,600 species of marine fishes^24–26^. Shorefish biodiversity is a critical resource to Filipinos, with the Philippines ranking eleventh in global marine capture production^27^ and Philippine fish markets are some of the most species diverse markets in the world^10^. Roughly 70% of Filipino nutritional protein comes from fishes and more than 1.6 million people depend on the fishing industry for their livelihood^28^. Not all fishes caught are sold within the country; the Philippines exported $46 million in fish filets and other fish meat in 2021 and 21% of that was imported by the United States^29^. Given the remarkable biodiversity of the region and the high species diversity sold in markets, accurate identification of seafood is essential to food safety and management for both the Philippines and the countries that import fish products from the Philippines. To inventory species sold in Philippine fish markets and additional diversity of the region, the Bureau of Fisheries and Aquatic Resources-National Fisheries Research and Development Institute (BFAR-NFRDI), Department of Agriculture, Philippines, and the NMNH developed a collaboration in 2011 to generate a genetic reference library based on voucher specimens to quickly and efficiently identify fishes, regardless of whether they are whole or processed (e.g., filets, fishmeal).

**Fig. 1:**
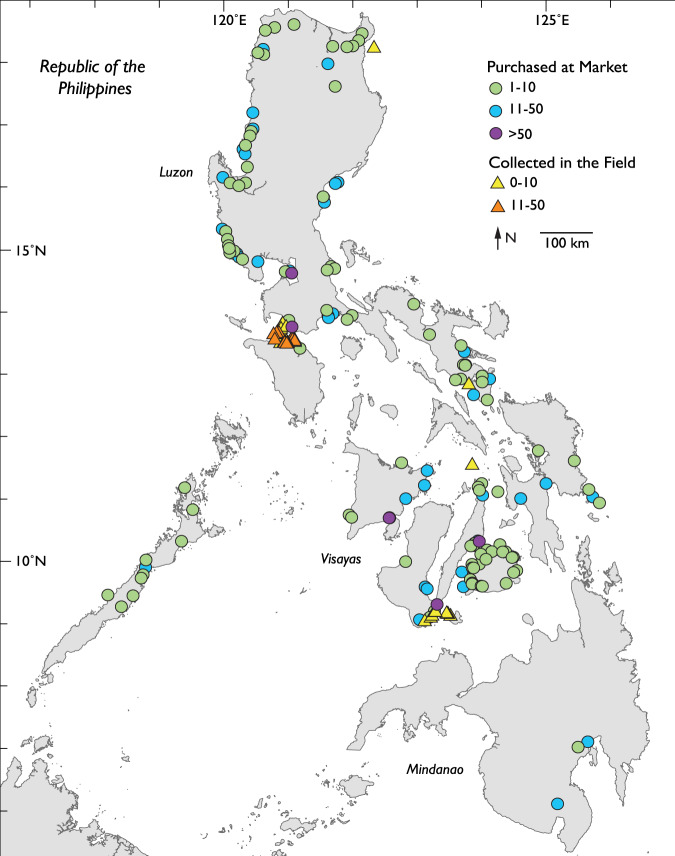
Map of sampling localities. For fishes purchased from markets, the market location is shown; vendors stated that fishes were captured from local coastal waters in the vicinity of the markets. For fishes collected in the field using rotenone while snorkeling, SCUBA diving, or using fishing gear, points on the map represent precise collection localities.

**Fig. 2:**
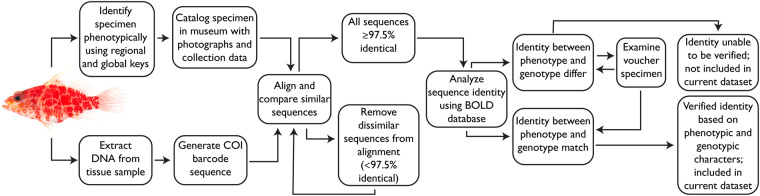
Methods for sequence identification and verification. Photograph of *Plectranthias inermis* USNM 431978, 33 mm SL.

**Fig. 3:**
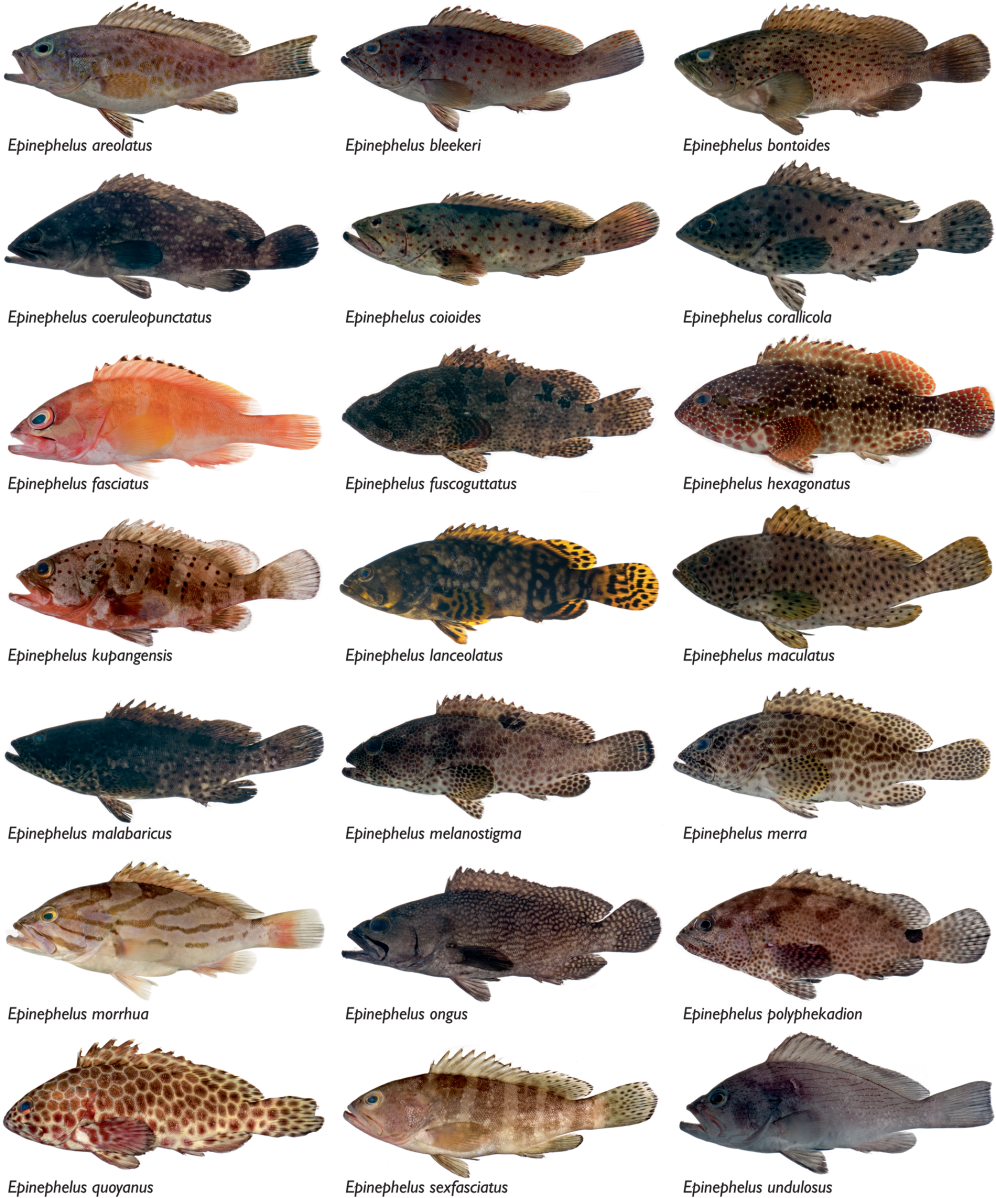
Photographs of groupers (*Epinephelus*), a challenging genus to identify and one that is frequently mislabeled. Live-color photographs, voucher specimens, and molecular sequence data enabled verified identifications of these specimens collected from Philippine fish markets. *Epinephelus areolatus* USNM 435700, 163 mm SL; *E. bleekeri* USNM 443235, 209 mm SL; *E. bontoides* USNM 403254, 160 mm SL; *E. coeruleopunctatus* USNM 431516, 223 mm SL; *E. coioides* USNM 435656, 237 mm SL; *E. corallicola* USNM 431624, 143 mm SL; *E. fasciatus* USNM 403060, 108 mm SL; *E. fuscoguttatus* USNM 431625, 261 mm SL; *E. hexagonatus* USNM 443283, 152 mm SL; *E. kupangensis* USNM 443558, 172 mm SL; *E. lanceolatus* USNM 431627, 204 mm SL; *E. maculatus* USNM 431623, 250 mm SL; *E. malabaricus* USNM 431611, 261 mm SL; *E. melanostigma* USNM 431622, 204 mm SL; *E. merra* USNM 443288, 144 mm SL; *E. morrhua* USNM 403199, 252 mm SL; *E. ongus* USNM 435567, 170 mm SL; *E. polyphekadion* USNM 423651, 154 mm SL; *E. quoyanus* USNM 445240, 215 mm SL; *E. sexfasciatus* USNM 445239, 151 mm SL; and *E. undulosus* USNM 431568, 181 mm SL.

**Fig. 4:**
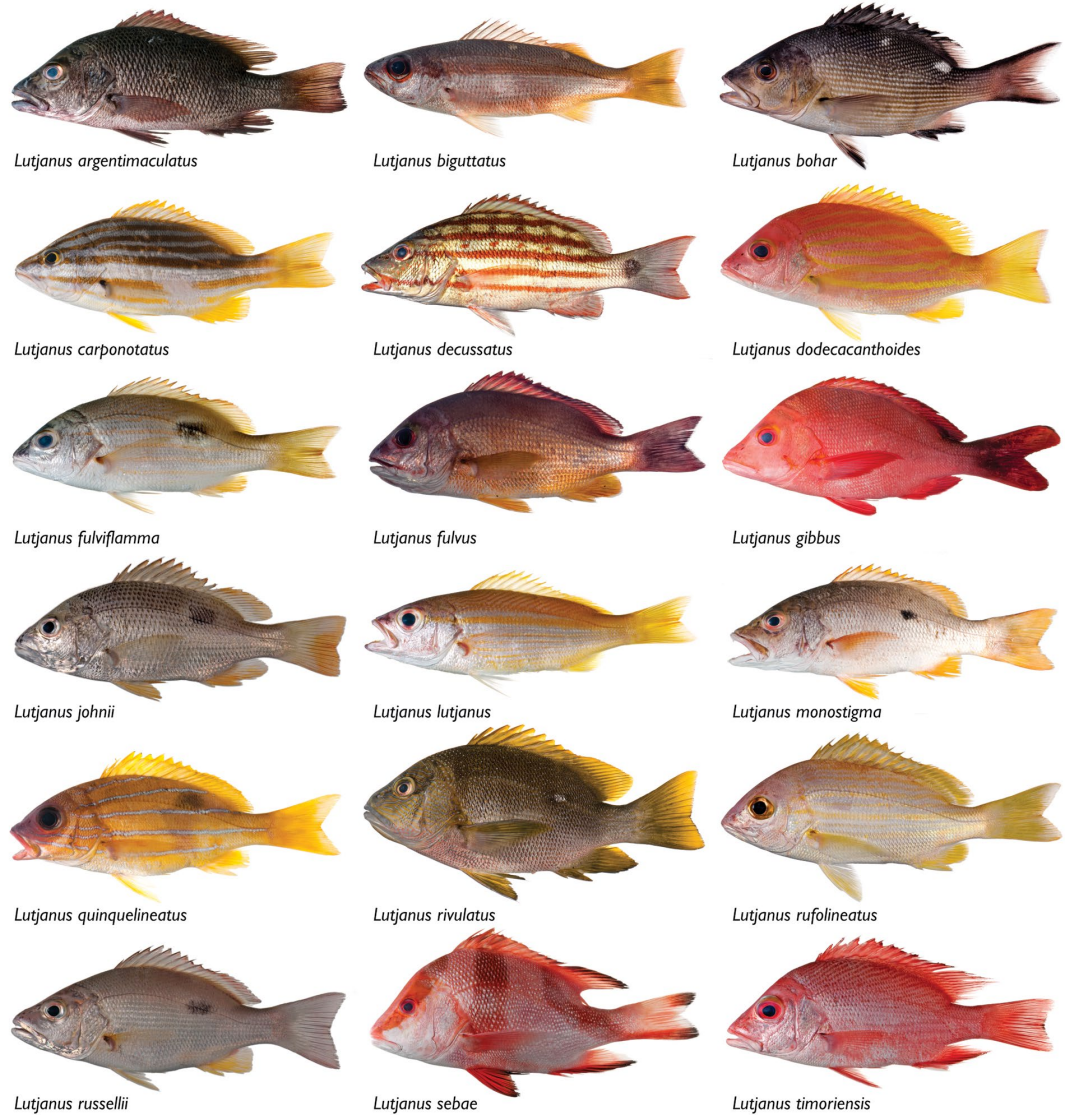
Photographs of snappers (*Lutjanus*) a commercially important genus; live-color photographs, voucher specimens, and molecular sequence data enabled verified identifications of these specimens collected from Philippine fish markets. *Lutjanus argentimaculatus* USNM 403082, 182 mm SL; *L. biguttatus* USNM 443245, 145 mm SL; *L. bohar* USNM 445453, 185 mm SL; *L. carponotatus* USNM 424827, 110 mm SL; *L. decussatus* USNM 445218, 170 mm SL; *L. dodecacanthoides* USNM 443545, 142 mm SL; *L. fulviflamma* USNM 403054, 134 mm SL; *L. fulvus* USNM 435687, 166 mm SL; *L. gibbus* USNM 403099, 223 mm SL; *L. johnii* USNM 423648, 139 mm SL; *L. lutjanus* USNM 403104, 127 mm SL; *L. monostigma* USNM 403313, 168 mm SL; *L. quinquelineatus* USNM 423610, 149 mm SL; *L. rivulatus* USNM 438092, 205 mm SL; *L. rufolineatus* USNM 445298, 148 mm SL; *L. russellii* USNM 423649, 144 mm SL; *L. sebae* USNM 403135, 256 mm SL; and *L. timoriensis* USNM 403119, 160 mm SL.

**Fig. 5:**
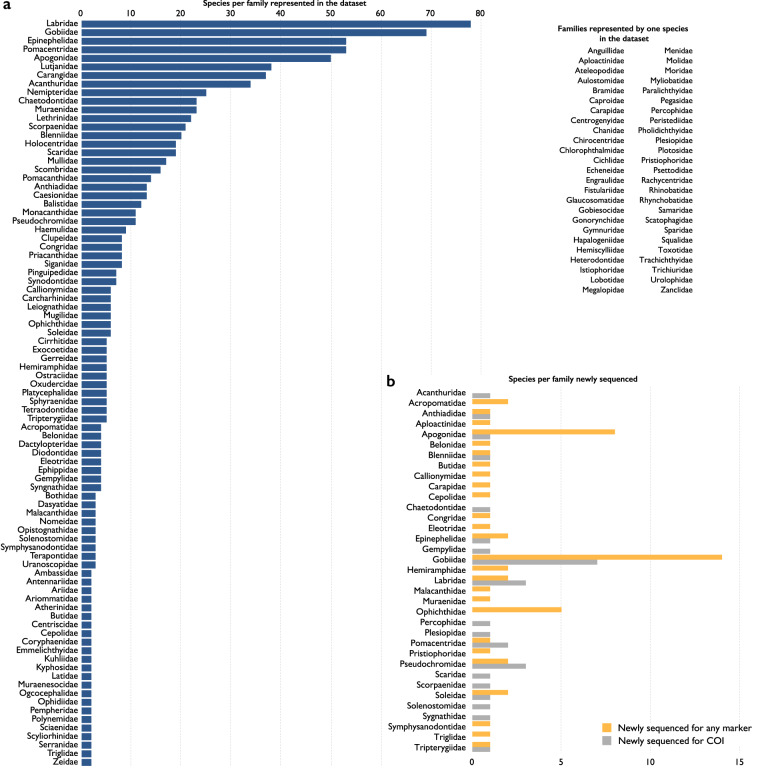
Species diversity included in the Biodiversity of Philippine Marine Fishes dataset. (**a**) Number of species by family; families represented by a single species shown to the right of the graph. (**b**) Number of newly sequenced species within each family in the dataset. Gold bars represent species publicly available for the first time; gray bars represent species for which some genetic data are available publicly but are first publicly available herein for COI.

Our dataset, Biodiversity of Philippine Marine Fishes, is the result of nine years (2011–2019) of sampling (Fig. 1) and includes 2,525 specimens representing 984 species. Seventy-seven percent of specimens in our dataset were purchased from fish markets; the remaining 23% were collected using rotenone or fishing gear to capture additional diversity of the region (Table 1). Specimens were sequenced for a ~655 base pair portion of the mitochondrial cytochrome c oxidase I locus (COI) to develop a barcode reference library linked to voucher specimens (Fig. 2). The dataset represents the most comprehensive DNA reference library of Philippine marine fishes, and includes vouchered museum specimens, collection data, live color photographs (e.g., Figs. 3, 4), and genetic samples for future analyses. Species identifications were verified based on taxonomic, phenotypic, and genotypic data (see Methods and Technical Validation sections; Fig. 2). Among the 135 families of fishes included in the verified dataset, the families Labridae (78 species), Gobiidae (69 species), Epinephelidae (53 species), and Pomacentridae (53 species) have the greatest species diversity (Fig. 5a). The dataset includes sequences for 55 species in 25 families that were not previously publicly available on GenBank or BOLD for any loci, and an additional 29 species in 19 families that represent the first publicly available COI barcode sequence for the taxon (as of September 28, 2022, Table 1, Fig. 5b; see Verified specimen records deposited at FigShare^30^). The Biodiversity of Philippine Marine Fishes dataset represents ~50% of the estimated Philippine market fish diversity^10^ and will serve as a foundational checklist and reference library to improve knowledge of species diversity and distributions, to identify and describe new species, and to confirm the identity of commercially caught fishes in this global biodiversity hotspot.

**Table 1:** Overview of 2,525 verified specimens in the dataset highlighting that different collecting methods are important for completing reference libraries. Most, 760 species, were purchased from markets, 340 species were collected in the field, and 116 species were collected both from the market and field. The majority of the newly sequenced (60%) and newly sequenced for COI species (69%) were collected in the field, likely because market collections emphasized commercial species with large maximum sizes, whereas less well-known cryptobenthic fishes were more often collected in the field.

	Purchased from market	Collected in field by rotenone or fishing gear	Combined datasets
Number of specimens	1,956 (77% of samples)	569 (23% of samples)	2,525
Number of species	760	340	984
Top three families for species diversity	Labridae, Epinephelidae, Lutjanidae	Gobiidae, Labridae, Pomacentridae	Labridae, Gobiidae, Epinephelidae
Number of newly sequenced species	22 (40%)	33 (60%)	55
Number of newly sequenced species for COI	9 (31%)	20 (69%)	29

## Methods

### Specimen collection

Between 2011 and 2019, we collected and verified 2,525 specimens, representing 135 families, 445 genera, and 984 species of fishes (see Verified specimen records deposited at FigShare^30^). Of these, 1,956 (77%) were purchased from fish landings, roadside stalls, and municipal and city markets (155 localities; Fig. 1; see Verified specimen records deposited at FigShare^30^). The remaining 569 fishes included in the dataset were collected from near-shore habitats using rotenone while snorkeling or SCUBA diving, or using fishing gear (71 localities; Fig. 1; see Verified specimen records deposited at FigShare^30^. Specimens were photographed in the field to capture live colors by J. T. Williams using Fujifilm and Nikon camera bodies with 105 mm macro lenses under flash or LED daylight lighting. Tissue samples from each specimen were preserved in ethanol and M2 lysis buffer (AutoGen Inc.), and whole voucher specimens were fixed using 10% formalin. Voucher specimens were transferred to 75% EtOH for long-term storage and cataloged in the NMNH Fish Collection and associated tissues and extractions were archived in the NMNH Biorepository (see Verified specimen records deposited at FigShare^30^. Specimen lengths used herein are reported as standard length (SL).

### Specimen identification and validation

Morphological identifications were made in the field on fresh specimens, and preserved specimens further examined at NMNH by J. T. Williams, K. E. Carpenter, K. E. Bemis, M. G. Girard, and D. E. Pitassy using global, regional, or taxon-specific keys (e.g.^25,31,32^). Voucher identities were verified using molecular characters by comparing newly generated sequences with those available through public repositories (e.g., GenBank, BOLD) as described in the Technical Validation section below and in Fig. 2. To obtain DNA sequence data, tissues were extracted using the AutoGenPrep 965 (AutoGen, Holliston, Massachusetts, USA) following manufacturer protocols. We targeted the 655 base-pair (bp) barcode of COI following Weigt *et al*.^7^ using the primers from Baldwin *et al*.^33^. Sequencing of PCR products was done on an Automated ABI 3730xl at the NMNH, with sequence trace files trimmed of low-quality ends and forward and reverse reads assembled into contigs using Sequencher 5.4 (Gene Codes). Resulting sequences were 515–686 bps in length (mean: 651.7, median: 655, mode: 655, standard deviation: 12.7). Sequence alignments were performed using MAFFT 7.475^34^ within Geneious 11.1.5^35^ (see Technical Validation section). Once sequences were confirmed (see Technical Validation section), taxonomic names were validated using either Eschmeyer’s Catalog of Fishes^36^ or FishBase^37^ using the ‘rfishbase’ 4 module^38^ within R 4.2.1.

## Data Records

The verified COI sequence library for Biodiversity of Philippine Marine Fishes includes (1) voucher specimens (2), tissues samples and DNA extracts (3), voucher collection information (4), live-color photographs, and (5) COI sequences of at least 500 bp. All photographs, voucher catalog numbers, DNA sequences, and collection data are publicly available through FigShare^30^. Data is also available on BOLD^39^, GenBank (BioProject PRJNA947503^40^), through the Fish Collection at the National Museum of Natural History Smithsonian Institution (https://collections.nmnh.si.edu/search/fishes/), and the FDA Reference Standard Sequence Library for Seafood Identification (RSSL; https://www.fda.gov/food/dna-based-seafood-identification/reference-standard-sequence-library-seafood-identification-rssl). The library follows the BARCODE data standard requirements^41,42^ for (1) species name (2), voucher data (3), collection data (4), sequence length (5), PCR primers used to generate the amplicon, and (6) trace files.

## Technical Validation

In addition to morphological identification, all vouchers were validated based on molecular characters using COI data from BOLD. The BOLD database was used for verification because it has more barcode sequences (when including those that are “private”) than available on GenBank and because barcodes published on GenBank are actively extracted and included within the BOLD database. Sequences generated from voucher specimens were imported into Geneious and multiple-sequence alignments were generated using MAFFT for each morphologically identified taxon. All taxon-specific alignments were checked individually to ensure each represented a single taxon and to identify divergent sequences and associated vouchers. Sequences were considered to be the same taxon if sequence identity was ≥97.5%. Sequences with similarity ≤97.4% were aligned with additional sequences and vouchers were examined to determine a revised identification.

Once each multiple-sequence alignment represented a single taxon based on our criteria, a representative sequence was submitted to the BOLD SYSTEMS Identification Engine via the web portal and searched against “All Barcode Records on BOLD.” The results, including the 101-terminal Neighbor-Joining (NJ) phylogeny, were examined to confirm identity of submitted sequences based on monophyletic groups. In total, 2,371 of the 2,525 (93.9%) sequences in our dataset were confirmed using publicly available sequences (see Verified specimen records deposited at FigShare^30^). For the remaining 154 sequences, representing 84 species, a matching COI barcode was not publicly available on GenBank or BOLD. These 154 sequences represent the first publicly available barcode for their identified taxon. Sequences in this category are either “Newly sequenced” or “Newly sequenced for COI” based on if they have been sequenced for one or more locus previously (Fig. 5b, see Verified specimen records deposited at FigShare^30^); determinations as of 28 September 2022).

## Usage Notes

The Biodiversity of Philippine Marine Fishes dataset is freely available to use for DNA barcoding or metabarcoding surveys, specimen identification, or other purposes (see Data Records). Additional specimens were collected that could not be verified based on currently available taxonomic, phenotypic, and genotypic information. Although not included in the verified sequence dataset released herein, these additional voucher specimens all have associated collection metadata, live-color photographs, and archived genetic samples available for future analyses (see Unverified specimen records deposited at FigShare^30^). As our understanding of the taxonomy of fishes in the Philippines increases, additional sequences from these collections will be verified and incorporated into the GenBank and BOLD projects.

## Data Availability

No custom code was used.
